# Are differences in stage at presentation a credible explanation for reported differences in the survival of patients with colorectal cancer in Europe?

**DOI:** 10.1054/bjoc.2001.1958

**Published:** 2001-09

**Authors:** C B J Woodman, A Gibbs, N Scott, N Y Haboubi, S Collins

**Affiliations:** Centre for Cancer Epidemiology, University of Manchester, Christie Hospital NHS Trust, Kinnaird Road, Withington, Manchester, M20 4QL; Department of Surgery, Hope Hospital; Department of Pathology, Withington Hospital, Manchester, UK

**Keywords:** colorectal cancer, survival, stage, EUROCARE

## Abstract

Popular reporting of a comparison of cancer survival rates across 17 European countries, based on data collected by national and regional cancer registries, has left an impression of inadequate treatment of patients in the UK. A subsequent study has suggested that the poor survival rates reported for the UK can, in large part, be explained by more advanced stage at presentation. We believe this conclusion to be unsound and use this study as an example to illustrate the methodological difficulties which may arise during such international comparisons. As the NHS cancer plan aspires to achieve for the UK parity with the best cancer care in Europe, careful thought needs to be given to identifying countries with which the UK can usefully compare itself and the most appropriate indicators for this comparison. http://www.bjcancer.com © 2001 Cancer Research Campaignhttp://www.bjcancer.com


*British Journal of Cancer* (2001) **85**(6), 787–790
© 2001 Cancer Research Campaign
doi: 10.1054/ bjoc.2001.1958, available online at http://www.idealibrary.com on IDEAL
http://www.bjcancer.com

### Are differences in stage at presentation a credible explanation for reported differences in the survival of patients with colorectal cancer in Europe?

**CBJ Woodman[1], A Gibbs[1], N Scott[2], NY Haboubi[3] and S Collins[1]**

[1]Centre for Cancer Epidemiology, University of Manchester, Christie Hospital NHS Trust, Kinnaird Road, Withington, Manchester M20 4QL; [2]Department of Surgery, Hope Hospital; and [3]Department of Pathology, Withington Hospital, Manchester, UK

**Summary** Popular reporting of a comparison of cancer survival rates across 17 European countries, based on data collected by national and regional cancer registries, has left an impression of inadequate treatment of patients in the UK. A subsequent study has suggested that the poor survival rates reported for the UK can, in large part, be explained by more advanced stage at presentation. We believe this conclusion to be unsound and use this study as an example to illustrate the methodological difficulties which may arise during such international comparisons. As the NHS cancer plan aspires to achieve for the UK parity with the best cancer care in Europe, careful thought needs to be given to identifying countries with which the UK can usefully compare itself and the most appropriate indicators for this comparison. © 2001 Cancer Research Campaign http://www.bjcancer.com

**Keywords**: colorectal cancer; survival; stage; EUROCARE

The NHS Cancer Plan asserts that ‘patients in the UK tend to have more advanced disease by the time their treatment begins than their counterparts in other European countries. This is thought to explain at least some of the poorer survival rates seen for some cancers in the UK’. Although no bibliography is provided with the NHS Cancer Plan, these observations are clearly based on the findings of the ‘high-resolution’ studies undertaken as part of the EUROCARE project; these studies, which collected detailed information on patient and disease related prognostic factors, were intended to provide an explanation for the survival differentials reported in EUROCARE II, a report which has contributed to an impression of inadequate care of cancer patients in the UK (Berrino et al, 1999). The first of these high-resolution (HR) studies, which describes the survival of patients with colorectal cancer diagnosed during 1989–91, has now been published, with the following conclusion: ‘the wide differences across Europe in colorectal cancer survival depend to a large extent on differences in stage at diagnosis. There are wide variations in diagnostic and surgical practices. There was a twofold range in the risk of death from colorectal cancer even after adjustment for surgery and disease stage’ (Gatta et al, 2000).

As the NHS Cancer Plan places considerable importance on achieving parity with the best cancer care in Europe, a detailed critique of the methodological problems revealed by this study, from the perspective of the UK, would appear timely.

*Received 3 January 2001*
*Revised 27 April 2001*
*Accepted 1 June 2001*

*Correspondence to:* CBJ Woodman

### Representativeness of cases included in the high resolution study

We first consider how representative of EUROCARE II are the countries included in the HR study. Whereas 47 cancer registries in 17 countries contributed to EUROCARE II, only 11 registries in 6 countries were included in the HR study. It is not clear how registries were identified for inclusion: no Scandinavian registry contributed cases, nor did any in Austria, Switzerland or Germany, countries which had among the highest survival rates for colorectal cancer in EUROCARE II; the HR study also includes a Spanish registry (Granada) which did not contribute cases of colorectal cancer to EUROCARE II, but includes none of the four Spanish registries which did.

We next consider how representative of EUROCARE II are the sample of cases submitted to the HR study by each cancer registry. Each registry was asked to provide a sample of at least 200 consecutive cases: a total of 2270 cases diagnosed between 1989 and 1991 were submitted; Thames, the cancer registry with the largest catchment population included in this study, failed to contribute the stated minimum number, as did Granada.

The investigators address the issue of representativeness by comparing the mean age and survival of the cases provided for the HR study with the corresponding values in EUROCARE II.

For all but three registries, the survival estimate for the cohort submitted to the HR study was very similar to that submitted to EUROCARE II (Table 1); the more substantial differences observed for the Mersey (44% vs 37%) and Rotterdam (48% vs 56%) cohorts are attributed to random variation, although the results of significance testing are not presented.

The 3-year survival rate reported for Modena (59%) in the HR study merits detailed consideration because it is the highest for any of the registries included in this study and is substantially different from that reported for the same registry in EUROCARE II (47%); we infer from the absence of any comment on random variation that in this case the difference *is* statistically significant.

**Table 1** Comparison of observed survival at 3 years for registries participating in the high resolution (HR) and EUROCARE II studies

| Registry | Observed survival at 3 years (%) HR study | EUROCARE II |
|---|---|---|
| Varese | 49 | 50 |
| Modena | 59 | 47 |
| Calvados | 53 | 47,45,50[1] |
| Somme | 50 | |
| Côte d'Or | 50 | |
| Rotterdam | 48 | 56 |
| Eindhoven | 55 | 51 |
| Granada | 46 | n/a[2] |
| Mersey | 44 | 37 |
| Thames | 38 | 40 |
| Cracow | 25 | 22 |

[1]Registries not separately identified in source; [2]Registry not included in EUROCARE II; Source: Gatta G et al (2000) *Gut* **47**: 533–538.

By way of explanation, the authors assert that the analysis in the HR study was confined to cases from the town of Modena, whereas the analysis reported in EUROCARE II included cases from the province of Modena. This explanation must be questioned because the Modena cancer registry, which covers the province of Modena with its population of 660 000, was only established in 1988 (Parkin et al, 1997). In EUROCARE II, however, Modena is listed as providing colorectal cases for the 5-year period 1984–89. It is difficult to avoid the conclusion that the cases contributed to both the EUROCARE II report and the HR study were not from the town and province of Modena respectively, as suggested, but were instead from the Modena colorectal cancer registry, established in 1984 in District 16 of the Region Emilia–Romagna; this district includes Modena and 10 smaller towns with a total population of 262 000 residents, which is almost identical in size to the reference population cited in EUROCARE II (Ponz de Leon et al, 1993).

A reanalysis of the tabulated data presented in EUROCARE II and in the HR study reveals a possible alternative explanation for the difference in survival between the Modena cohorts included in these reports: compared with the EUROCARE II cohort there was a significantly lower mortality rate at 1 month after diagnosis in the HR study (2% vs 5%, Fisher's exact test $P = 0.016$). Were this to reflect a failure to include in the HR cohort all patients with advanced disease, it might provide an explanation for the extremely high survival at 3 years observed in this cohort for both unresected cases (25%) and for those with advanced disease (25%).

The HR study also suggests that medical care may be better in Modena than elsewhere in Italy; this may be the case, but among the Italian registries, Modena did not have the best survival rates for either colon or rectal cancer either in EUROCARE II or in the cited study on regional differences in cancer survival in Italy (Gatta et al, 1997).

We have some reservations about the representativeness of cases submitted to the HR study by the two English registries. The authors report that the 3-year survival of cases contributed to EUROCARE II was 40% for Thames and 37% for Mersey; in contrast, for cases included in the HR study, residents of the Mersey region with colorectal cancer had a better survival than those from the Thames region (44% vs 38%). Furthermore, compared with the Thames cohort of colorectal cancers included in EUROCARE II, that in the HR study had significantly more deaths within one month of diagnosis (16% vs 9%, Fisher's exact test $P = 0.0029$).

In addition, a previous study carried out within the EUROCARE network, reported surgical resection rates for all colorectal cancers incident in 1987 in 15 European populations, for whom relatively complete data on treatment were available; 8 of the registries participating in this earlier study also contributed cases to the HR analysis (Gatta et al, 1996). With the exception of Cracow and Thames, surgical resection rates are higher in the HR than in the earlier study; the largest increase was observed for cases contributed by the Mersey registry, from 66% to 82%. A formal statistical comparison is not possible because the earlier study reports an age adjusted rate and the latter a crude rate; the difference, however, is unlikely to be explained by age adjustment because the more recent Mersey cohort includes a greater proportion of cases aged 75 years and over. It is also surprising, given the general impression of inadequate care of cancer patients in the UK left by the EUROCARE reports, that in the HR study the proportion of patients who underwent surgery and who had this as an emergency procedure was lowest in the sample contributed by the Mersey registry.

### Comparison of survival rates

When estimating survival from population based data it is not always possible to decide from death certificates whether a patient has died from cancer or from an unrelated cause. To overcome this problem it is usual to include all deaths in the analysis but to allow for other causes of death by calculating the relative survival rate, defined as the ratio of the observed survival rate to the survival rate which would be expected in an underlying population similarly constituted with respect to age and sex.

In the HR study, the investigators chose to present observed rather than relative survival rates. EUROCARE II reports both observed and relative survival rates, and the more detailed site-specific reports based on EUROCARE II data which appeared in the European Journal of Cancer present relative survival rates (Gatta et al, 1998). The authors do state that multiple regression analysis of relative survival rates, the conventional analysis in this situation, was carried out, but only to check the proportional hazards analysis presented. The authors justify the presentation of observed rather than relative survival rates by arguing that at 3 years, competing causes of mortality are likely to have a minimal impact on survival estimates; and present the minimum (1.4%) and maximum (1.8%) differences between observed and relative survival estimates for registries included in this analysis.

**Table 2** A comparison of the observed and relative survival rates at 3 years for colon cancer and cancer of the rectum reported in EUROCARE II

| | Colon | | Rectum | |
|---|---|---|---|---|
| | 3-year observed survival (%) | 3-year relative survival (%) | 3-year observed survival (%) | 3-year relative survival (%) |
| Italy | 46 | 53 | 47 | 53 |
| France | 50 | 58 | 51 | 58 |
| Netherlands | 54 | 62 | 53 | 60 |
| Spain | 47 | 53 | 44 | 49 |
| England | 39 | 46 | 41 | 48 |
| Poland | 25 | 29 | 27 | 31 |

 *© 2001 Cancer Research Campaign*

The modesty of these differences is surprising because a comparison using EUROCARE II data, of the observed and relative survival rates at 3 years for patients with colon and rectal cancer, in countries which also contributed cases to the HR study, reveals substantially greater differences (Table 2). Reasons for this disparity are not immediately obvious.

### Interpretation

When considering the interpretation of these results, it should be pointed out that considerable caution must be exercised when comparing stage-specific survival rates across registries, when not only does the proportion of unstaged cases vary from 4% to 39%, but the survival of unstaged cases also varies substantially, from 8% to 54%. The conflation of Dukes' stage A and B tumours is counterintuitive, given that these stages have different survival rates. The authors' characterization of both Dukes' stage A and B tumours as being confined to the bowel wall, is inconsistent with the classification system proposed by Dukes (Dukes, 1932).

The authors of the HR study conclude that 'across Europe survival differences were large but narrowed when corrections for stage were applied.' Were the result for Cracow, an outlier in almost all respects, ignored, then the relative risk of death compared to Varese (reference category) adjusted for age, sex and site but prior to adjustment for stage varied across the remaining registries from 0.73 to 1.41, and after adjustment for stage, from 0.76 to 1.37. Inspection of the range of relative risks before and after adjustment for an explanatory variable is a simplistic method for considering the amount of variation thus explained, but even using this method, the effect of stage does not appear to be great.

The authors also suggest that after appropriate correction for stage, '...in the Thames region, survival became similar to the other Western European registries, suggesting that the main reason for the low survival in the Thames area was late stage at diagnosis.' This is demonstrably not the case: after controlling for age, sex and site, further adjustment for stage had a minimal impact on the relative risk of death for cases contributed by the Thames registry (from 1.41 to 1.37); this remained statistically significant. The authors conclude that 'the large differences in the survival of unresected patients [0% to 25% three year survival] most likely reflect different therapeutic approaches to patients with little chance of being cured.' We disagree. We would of course like to believe that the 25% survival rate at 3 years achieved in unresected patients in Modena is attainable elsewhere, but are not convinced that such an outcome was plausible during the period when these cases were diagnosed. We would suggest that either misdiagnosis or a failure to ascertain all deaths is a more likely explanation. The final model presented in the HR study does suggest that with the exception of Cracow, there is no significant difference in outcome in surgically resected cases.

### DISCUSSION

The high-resolution study has substantial methodological flaws and provides no convincing evidence that stage at diagnosis can explain, for colorectal cancer at least, the differences in survival between the UK and other countries in Europe.

The lack of consistency across the different publications from the EUROCARE project is particularly disturbing because the NHS Cancer Plan promises 'the fastest improvement in cancer services anywhere in Europe over the next five years. By 2010, our five year survival rates for cancer will compare with the best in Europe.' This promise may offer a hostage to fortune because, as we have previously argued, for some cancer registries which contributed to EUROCARE II, survival rates were likely to be inflated because of the failure to register all cases of advanced disease and/or the failure to ascertain deaths in registered cases (Prior et al, 1998; Moran et al, 2000). Furthermore, whereas there is complete coverage of cancer registration in the UK, many of the countries contributing to EUROCARE II were represented by registries which covered only a fraction of their total population. The authors of EUROCARE II concede that their experience may not be entirely representative of cancer survival in those countries (Berrino et al, 1999).

Careful thought therefore needs to be given to identifying countries with which the UK can usefully compare itself. Ideally, these countries should have mature cancer surveillance programmes with total population coverage, systems in place for the collection and collation of data from multiple sources in order to maximize case ascertainment, and access and efficient linkage to death certificates; the Scandinavian countries fulfil all of these criteria and all, with the exception of Denmark, have better survival rates than the UK for many sites of cancer.

However an assessment of the progress made by the UK towards convergence with its European comparators should not wait until 5-year cancer survival rates become available in 2010. Sub-optimal care needs to be promptly identified and rectified; those lessons which can be learnt from comparators need to be learnt as soon as possible.

One way forward would be to focus population-based comparisons on those processes of care which can be clearly linked to outcome, for example the use of adjuvant chemotherapy in the management of patients with bowel cancer. It should be noted that for those sites of cancer for which some countries have established screening programmes, for example breast cancer, comparisons based on cancer survival rates can be misleading.

A rolling audit programme based on genuinely representative samples of cases would provide more useful information on how the delivery of cancer services in the UK might be improved. Such a development might be timely, given the improvement in completeness of information on stage of disease routinely captured by the UK registries, and the proposed expansion of the minimum dataset which they collect. For the first time it would also provide a credible population-based comparison of the distribution of patient and disease related prognostic factors in countries with a range of cancer survival rates; this intelligence is critical to the future development of the UK cancer control strategy.

### REFERENCES

Berrino F, Capocaccia R, Esteve J, Gatta G, Hakulinen T, Micheli A, Sant M and Verdecchia A (1999) *Survival of cancer patients in Europe: the EUROCARE-2 Study. IARC Scientific Publications No 151*. International Agency for Research on Cancer: Lyon

Coleman M, Babb P, Damiecki P, Grosclaude P, Honjo S, Jones J, Knerer G, Pitard A, Quinn M, Sloggett A and De Stavola B (1999) *Cancer survival trends in England and Wales, 1971–1995: deprivation and NHS Region. Studies in Medical and Population Subjects No 61*. The Stationery Office: London

Dukes C (1932) The classification of cancer of the rectum. *Journal of Pathology and Bacteriology* **35**: 323–332

Gatta G, Sant M, Coebergh JW, Hakulinen T and the EUROCARE working group (1996) Substantial variation in therapy for colorectal cancer across Europe:

© 2001 Cancer Research Campaign 

EUROCARE analysis of cancer registry data for 1987. *Eur J Cancer* **32A**: 831–835

Gatta G, Buiatti E, Conti E, De Lisi V, Falcini F, Federico M, Gafà L, Ponz de Leon M, Vercelli M and Zanetti R (1997) Variations in the survival of adult cancer patients in Italy. *Tumori* **83**: 497–504

Gatta G, Faivre J, Capocaccia R, Ponz de Leon M and the EUROCARE working group (1998) Survival of colorectal cancer patients in Europe during the period 1978–1989. *Eur J Cancer* **34**: 2176–2183

Gatta G, Capocaccia R, Sant M, Bell CMJ, Coebergh JWW, Damhuis RAM, Faivre J, Martinez-Garcia C, Pawlega J, Ponz de Leon M, Pottier D, Raverdy N, Williams EMI and Berrino F (2000) Understanding variations in survival for colorectal cancer in Europe: a EUROCARE high-resolution study. *Gut* **47**: 533–538

Moran A, Collins S, Gibbs A and Woodman CBJ (2000) Survival of patients with colon cancer in Europe: a cautionary tale. *Colorectal Disease* **2**: 190–192

Parkin D, Whelan S, Ferlay J, Raymond L and Young J (1997) *Cancer Incidence in Five Continents Volume VII. IARC Scientific Publications No 143*. International Agency for Research on Cancer: Lyon

Ponz de Leon M, Sassatelli R, Scalmati A, Di Gregorio C, Fante R, Zanghieri G, Roncucci L, Sant M and Micheli A (1993) Descriptive epidemiology of colorectal cancer in Italy: The 6 year experience of a specialised registry. *Eur J Cancer* **29A**: 367–371

Prior P, Woodman CBJ and Collins S (1998) International differences in survival from colon cancer: more effective care versus less complete cancer registration. *Br J Surg* **85**: 101–104

 *© 2001 Cancer Research Campaign*
